# Predictive Models for Kidney Recovery and Death in Patients Continuing Dialysis as Outpatients after Starting in Hospital

**DOI:** 10.2215/CJN.0000000000000173

**Published:** 2023-04-18

**Authors:** Edward G. Clark, Matthew T. James, Swapnil Hiremath, Manish M. Sood, Ron Wald, Amit X. Garg, Samuel A. Silver, Zhi Tan, Carl van Walraven

**Affiliations:** 1 Division of Nephrology, Department of Medicine, University of Ottawa, Ottawa, Ontario, Canada; 2 Kidney Research Centre, Ottawa Hospital Research Institute, Ottawa, Ontario, Canada; 3 Division of Nephrology, Department of Medicine, Cumming School of Medicine, University of Calgary, Calgary, Alberta, Canada; 4 Department of Community Health Sciences, Cumming School of Medicine, O'Brien Institute of Public Health, University of Calgary, Calgary, Alberta, Canada; 5 Department of Medicine and Epidemiology and Community Medicine, University of Ottawa, Ottawa, Ontario, Canada; 6 Institute for Clinical Evaluative Sciences, Toronto, Ontario, Canada; 7 Division of Nephrology, Department of Medicine, St. Michael's Hospital, University of Toronto, Toronto, Ontario, Canada; 8 Division of Nephrology, Department of Medicine, Western University, London, Ontario, Canada; 9 Lawson Health Research Institute, London Health Sciences Centre, London, Ontario, Canada; 10 Division of Nephrology, Department of Medicine, Queens University, Kingston, Ontario, Canada; 11 Department of Medicine, The Ottawa Hospital, University of Ottawa, Ottawa, Ontario, Canada

**Keywords:** acute renal failure, clinical epidemiology, dialysis, ESKD, outpatients, hospitals

## Abstract

**Background:**

For patients who initiate dialysis during a hospital admission and continue to require dialysis after discharge, outpatient dialysis management could be improved by better understanding the future likelihood of recovery to dialysis independence and the competing risk of death.

**Methods:**

We derived and validated linked models to predict the subsequent recovery to dialysis independence and death within 1 year of hospital discharge using a population-based cohort of 7657 patients in Ontario, Canada. Predictive variables included age, comorbidities, length of hospital admission, intensive care status, discharge disposition, and prehospital admission eGFR and random urine albumin-to-creatinine ratio. Models were externally validated in 1503 contemporaneous patients from Alberta, Canada. Both models were created using proportional hazards survival analysis, with the “Recovery Model” using Fine–Gray methods. Probabilities generated from both models were used to develop 16 distinct “Recovery and Death in Outpatients” (ReDO) risk groups.

**Results:**

ReDO risk groups in the derivation group had significantly distinct 1-year probabilities for recovery to dialysis independence (first quartile: 10% [95% confidence interval (CI), 9% to 11%]; fourth quartile: 73% [70% to 77%]) and for death (first quartile: 12% [11% to 13%]; fourth quartile: 46% [43% to 50%]). In the validation group, model discrimination was modest (c-statistics [95% CI] for recovery and for death quartiles were 0.70 [0.67 to 0.73] and 0.66 [0.62 to 0.69], respectively), but calibration was excellent (integrated calibration index [95% CI] was 7% [5% to 9%] and 4% [2% to 6%] for recovery and death, respectively).

**Conclusions:**

The ReDO models generated accurate expected probabilities of recovery to dialysis independence and death in patients who continued outpatient dialysis after initiating dialysis in hospital. An online tool on the basis of the models is available at https://qxmd.com/calculate/calculator_874.

## Visual Abstract

**Figure:**
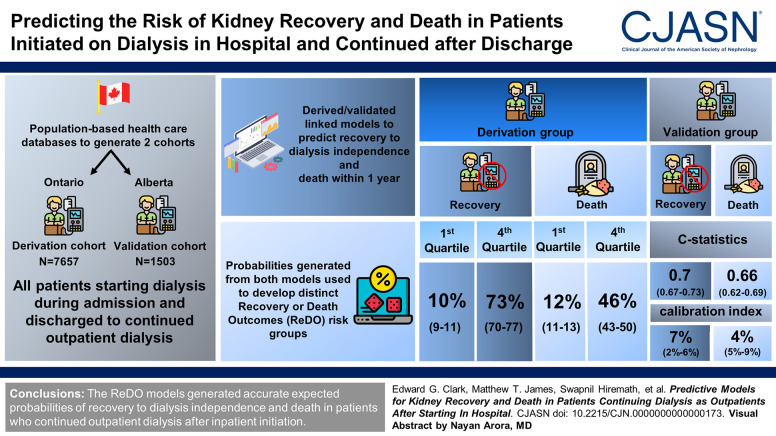

## Introduction

Dialysis is initiated in hospital for management of AKI or advanced CKD, and there can be significant overlap (“AKI on CKD”).^1^ AKI treated with dialysis (AKI-D) occurs in approximately 2% of all hospitalized patients.^2^ Approximately 50% of patients with AKI-D survive their index hospitalization, of who up to 30% remain dialysis dependent after discharge.^3,4^ These patients continue their dialysis treatments as outpatients, with approximately half recovering enough kidney function to discontinue dialysis within 90 days.^3^ After 90 days, only approximately 5% additional patients recover sufficient kidney function to stop dialysis. Of all patients with AKI-D who survive to discharge, approximately 35% die within 6 months,^5^ most commonly from cardiovascular disease.^3^ Patients may also start dialysis in hospital due to progressive CKD, but the extent to which superimposed AKI precipitated dialysis initiation can be difficult to determine, and the likelihood of kidney function recovery is highly variable. Prognostication is further complicated by worse outcomes when dialysis for kidney failure due primarily to CKD progression is initiated in hospital.^6^

No predictive models currently exist to estimate a patient's likelihood of kidney recovery to dialysis independence or death when discharged to continue dialysis after hospitalization. An improved understanding of these risks could improve outpatient dialysis management for this patient population. To help address this knowledge gap, we derived and externally validated risk indexes to predict the likelihood of recovery to dialysis independence and of death within 1 year after discharge for patients continuing outpatient dialysis after in-hospital dialysis initiation.

Such risk indexes could be applicable to patients who start dialysis in hospital at any level of prehospitalization kidney function (*i.e.*, due to AKI, “AKI on CKD”, or progression of CKD) and then continue to receive dialysis after discharge. Furthermore, they could be well suited to implementation using routine data available at the time of patients' first outpatient dialysis after hospital discharge.

## Methods

### Study Design

This study used linked population-based health care databases from the provinces of Ontario and Alberta, Canada, maintained by ICES (formerly known as Institute for Clinical Evaluative Sciences) and the Alberta Kidney Disease Network, respectively. Ontario and Alberta residents have universal access to government-funded hospital care, physician services, and both in-patient and out-patient dialysis. This study was approved by the Sunnybrook Health Sciences Research Ethics Board (Toronto, ON) and the University of Calgary Conjoint Health Research Ethics Board, both of which waived the requirement of patient consent because of the retrospective and anonymous nature of this study. This study is reported in accordance with the “Transparent Reporting for a Multivariable Prediction Model for Individual Prognosis Or Diagnosis”^7^ statement.

### Data Sources

The population-based datasets used for this study captured data for all people living in Ontario and Alberta, Canada (with populations in 2017 of 12.8 and 4.2 million, respectively). The datasets used for this study are presented in Supplemental Table 1. Codes used for this study are presented in Supplemental Table 2.

### Study Cohorts

Our derivation and external validation cohorts included all patients starting dialysis (that is, any form of KRT) during hospitalization from which they were discharged alive to continue outpatient dialysis. These cohorts were created by first identifying all hospital encounters between January 1, 2008, and March 31, 2018. These dates captured the time frame during which outpatient preadmission serum creatinine measures were available in Ontario. We then selected all hospitalizations during which patients were coded with acute dialysis procedures (Supplemental Table 2) and limited observations to each patient's first such hospitalization. We then excluded (*1*) patients who died before hospital discharge; (*2*) patients who did *not* continue dialysis after discharge from hospital, defined as no dialysis billing claims within 1 week after hospital discharge; (*3*) patients with one or more dialysis procedure(s) or kidney transplant before their index hospitalization; (*4*) patients with no outpatient serum creatinine measurements during the period 7–365 days before their index hospitalization; and (*5*) patients younger than 18 years.

### Outcomes

The two primary outcomes determined within 1 year after discharge from hospital were (*1*) kidney recovery to dialysis independence and (*2*) all-cause mortality. The 1-year time frame was chosen to facilitate timely decision making for fistula creation or transplantation assessment, and recovery after 1 year is rare.^5,8,9^ Patients were classified as having recovery to dialysis independence if they remained alive for at least 30 days without dialysis (either hemodialysis or peritoneal dialysis) or kidney transplant billing codes. When patients met these criteria, the date following their last dialysis code was considered the date they achieved recovery to dialysis independence. For patients with their last dialysis code within 30 days before the end of the observation period, we extended it to include 1 year plus 30 days past the last dialysis code to ensure they never continued/restarted dialysis. If neither of these events occurred, they were classified as dialysis-free at the time of their last dialysis code.

### Covariables

We determined each patient's demographic details (age, sex, and domicile status), health burden (Charlson score, use of home oxygen [in derivation population only], and new cancer diagnosed in previous 5 years), and acuity of illness (admission urgency and critical care unit status). We also determined whether patients had been classified as having congestive heart failure, acute myocardial infarction, or diabetes mellitus before their admission (Supplemental Table 2). Comorbidity burden was quantified using the Charlson score with coding criteria from Quan *et al.*^10^ and weights from Schneeweiss *et al.*^11^ Death risk was determined using the Hospitalized-patient One-year Mortality Risk score, a highly discriminative (c-statistic 0.89) and well-calibrated index that has been externally validated.^12^

We defined baseline serum creatinine as the most recent outpatient value 7–365 days before the index admission.^13^ Outpatient serum creatinine values in Ontario have previously been shown to have small measurement error.^14^ All laboratory results across Ontario and Alberta complied with isotope dilution mass spectrometry standards during the study period.^15^ Prehospitalization eGFR was determined using the Chronic Kidney Disease Epidemiology Collaboration equation without the coefficient for Black race.^16^ We also determined patients' prehospitalization urine albumin-to-creatinine ratios (ACRs), using those temporally closest to the serum creatinine used to estimate baseline eGFR.

### Analysis

We used the Ontario cohort to create two models to classify patients into “Recovery and Death in Outpatients” (ReDO) risk groups. The first model was a competing risk proportional subhazards model (using the methods of Fine and Gray^17^) to model time from hospital discharge to recovery to dialysis independence within 1 year accounting for the competing risk of death. The second model was a standard Cox proportional hazards model for time from hospital discharge to death from any cause within 1 year. Because we wanted parsimonious models with optimal utility and generalizability, we used forward variable selection to identify variables whose association with the outcome had a *P* value <0.001. We used fractional polynomial methods to identify the optimal transformations for continuous and count variables.^18,19^

Coefficients from both models were converted to integer values to create an integer-based risk index for each outcome (the Recovery Score and the Death Score, respectively) using methods described by Sullivan *et al.*^20^ Both scores were then categorized into quartiles having similar number of outcomes. Concurrently, assigning patients to a Recovery and a Death quartile resulted in 16 groups (“ReDO groups”). We used a 5000 bootstrap sample of the derivation cohort to return the expected 1-year risk (with 95% confidence intervals (CIs) using the percentile method^21^) using cumulative incidence functions for both outcomes in each ReDO group.

Recovery and death models were externally validated using the Alberta cohort. Discrimination was measured using the c-statistic. Calibration was measured using the Integrated Calibration Index (ICI).^22^ The latter statistic quantifies the weighted difference between observed and predicted probabilities, ranging from 0% (perfect agreement) to 100% (perfect disagreement). Calibration plots with intercept of observed versus expected outcome probabilities were generated using simple least squares regression weighted by the inverse variance of the observed proportion.

For missing data, urine ACR was treated as a categorical variable that included missing as a category. For all other variables, if a participant did not meet criteria for a particular categorical variable, it was considered absent.

## Results

### Cohort Characteristics

**Figure 1:**
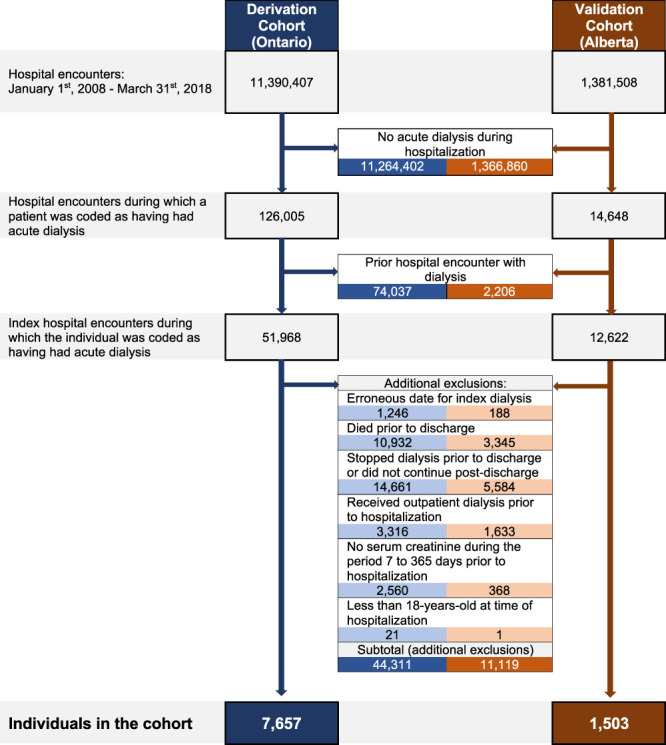
Creation of derivation and validation cohorts.

Creation of the derivation and external validation cohorts is presented in Figure 1. The derivation cohort had 7657 people (Table 1). Patients had a mean age of 68 years, and 62% were male patients. The median (interquartile range [IQR]) Charlson score was 5 (3–6). Preexisting congestive heart failure and diabetes mellitus were present in over half of the patients. Over half of the patients had baseline eGFR >15 ml/min per 1.73 m^2^, and approximately one quarter had baseline eGFR >30 ml/min per 1.73 m^2^. Urine ACR exceeded 30 mg/mmol in almost half of patients while almost a third of patients had no measure. Approximately one fifth of patients were admitted electively to hospital (21%), admitted directly to a critical care unit (19%), or underwent their initial dialysis in a critical care unit (20%).

**Table 1:** Characteristics of the derivation and validation cohorts of patients continuing outpatient dialysis after starting in hospital

Cohort	Derivation (Ontario) *n*=7657	Validation (Alberta) *n*=1503
Mean age (SD)	67 (14)	64 (15)
Male, No. (%)	4768 (62)	919 (61)
Diabetes mellitus, No. (%)	5140 (67)	895 (60)
Previous acute myocardial infarction, No. (%)	1177 (15)	280 (19)
Congestive heart failure, No. (%)	3969 (52)	595 (40)
Home oxygen, No. (%)	381 (5)	N/A
Median Charlson score (IQR)	5 (3–6)	5 (3–6)
**Cancer diagnoses****^a^** **in the past 5 yr, No. (%)**		
Any type	968 (13)	344 (23)
Lung	62 (0.8)	33 (2)
Breast	59 (0.8)	23 (2)
KUB	190 (2)	103 (7)
Myeloma	313 (4)	43 (3)
Prostate	76 (1)	55 (4)
Colorectal	105 (1)	35 (2)
Other	234 (3)	115 (8)
**Baseline eGFR in ml/min per 1.73 m** ^**2**^ **, No. (%)**		
<15	3840 (50)	771 (51)
15–30	1853 (24)	315 (21)
30–60	1022 (13)	204 (14)
>60	942 (12)	213 (14)
**Baseline Urine ACR, No. (%)**		
Not measured	2360 (31)	978 (65)
<3 mg/mmol	582 (8)	59 (4)
3–30 mg/mmol	1182 (15)	102 (7)
>30 mg/mmol	3533 (46)	364 (24)
Previous nephrology consultation, No. (%)	3321 (43)	999 (66)
Median % 1-yr death^b^ (IQR)	20 (9–37)	N/A
**Admission type, No. (%)**		
Elective	1611 (21)	111 (7)
Urgent, no ambulance	2761 (36)	355 (24)
Urgent, ambulance	3285 (43)	1037 (69)
Admitted to an intensive care unit, No. (%)	1425 (19)	414 (28)
30-d urgent readmission, No. (%)	1735 (23)	305 (20)
Median days from admission to dialysis initiation (IQR)	5 (2–12)	3 (1–7)
Dialysis first performed in ICU, No. (%)	1509 (20)	275 (18)
CKRT during index admission, No. (%)	596 (8)	212 (14)
Median days from dialysis initiation to hospital discharge (IQR)	9 (4–20)	9 (3–22)
**Discharge disposition, No. (%)**		
Independent	5381 (70)	827 (55)
Rehab	21 (0.3)	291 (19)
Home care	1988 (26)	277 (18)
Nursing home	214 (3)	108 (7)
CH	53 (0.7)	N/A

For Alberta data: (*1*) all necessary variables were not available to calculate risk of death at 1 year on the basis of Hospitalized-patient One-year Mortality Risk score; (*2*) discharge disposition to rehabilitation versus chronic hospital could not be distinguished; (*3*) values in the row for “Rehab” represent discharges to either a rehab or chronic hospital in Alberta. N/A, not available; IQR, interquartile range; KUB, kidney ureter or bladder; ACR, albumin-to-creatinine ratio; ICU, intensive care unit; CKRT, continuous KRT; CH, chronic hospital.

a Cancer diagnoses in the past 5 years on the basis of cancer registry data for Ontario and, for Alberta, discharge abstract database and physician claims.

b As predicted using the Hospitalized-patient One-year Mortality Risk.

The validation cohort included 1503 patients (Table 1). Notable differences from the derivation cohort included younger age (mean age 64 versus 68 years), a lower likelihood of congestive heart failure (40% versus 52%) or being discharged to the community (55% versus 70%), a higher likelihood of cancer (23% versus 13%), urgent admissions by ambulance (69% versus 43%), and unmeasured ACR values in the previous year (65% versus 31%). The breakdown according to baseline eGFR category in the validation cohort was similar to the derivation cohort, with 49% having a baseline eGFR >15 and 28% having a baseline eGFR >30. Similar to the derivation cohort, 18% of patients received initial dialysis in a critical care unit, albeit with more frequent use of continuous KRT (14% versus 8%).

Outcomes 1 year after hospital discharge were similar when the derivation and validation cohorts were compared: 1445 (19%) versus 316 (21%) patients were alive without dialysis, 1651 (22%) versus 316 (21%) had died, and 4551 (59%) versus 871 (58%) required ongoing dialysis (or had received a kidney transplant), respectively. Compared with those who died or those who did not recover to dialysis independence, patients alive without dialysis at 1 year were younger, had fewer comorbidities, had a higher preadmission eGFR, were more likely to have urine ACR <3 mg/mmol, and were more likely to be living independently after discharge (see Supplemental Table 3).

### Risk Models and Scores

**Figure 2:**
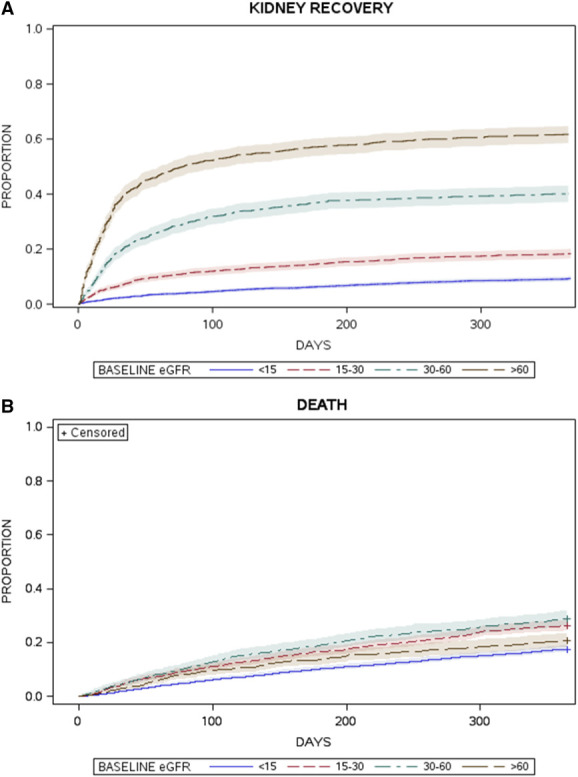
**Probabilities of kidney recovery and death vary according to baseline eGFR.** Cumulative incidence functions for kidney recovery to dialysis independence (A) and death (B) by days from hospital discharge in the derivation cohort according to baseline eGFR (before hospitalization). The probability (vertical axis) of kidney recovery to dialysis independence (A) and death (B) is presented by days after discharge from hospital (horizontal line). Probability estimates are flanked by 95% confidence intervals; were determined with cumulative incidence functions, which account for the competing risks; and are stratified by baseline eGFR (before hospitalization).

The 1-year likelihood of recovery to dialysis independence (accounting for the competing risk of death) in the derivation group was 24% (95% CI, 23% to 25%) (Figure 2A). The predicted probability of recovery was higher at higher levels of baseline kidney function. In the final recovery model (Supplemental Table 4), factors associated with higher predicted probability of recovery included lower Charlson score, less albuminuria at baseline, fewer days from admission to initial dialysis, fewer days from dialysis initiation to discharge, and initial dialysis in a critical care unit.

The 1-year death risk in the derivation group was 22% (95% CI, 21% to 23%). This risk also varied significantly by prehospitalization eGFR category but in a nonlinear fashion (Figure 2B); the predicted probability of death was highest in patients with preadmission eGFR 30–60 ml/min per 1.73 m^2^ and lowest for those with preadmission eGFR <15 ml/min per 1.73 m^2^. In the final death model (Supplemental Table 4), factors associated with a higher predicted probability of death included older age, greater comorbidity index, and diagnoses in the previous 5 years with cancers of lung, breast, or urogenital system as well as myeloma and other cancers (excluding prostate and colorectal cancers). Compared with those discharged to independent living situations, the predicted probability of death was higher in patients who required home nursing services after discharge or were discharged to nursing homes.

The ReDO-Kidney Recovery Score is presented in Table 2, and the ReDO Death Score is presented in Table 3. The ReDO-Kidney Recovery Score had a median value (IQR) of 6 (0–16) and the c-statistic was 0.73 (95% CI, 0.69 to 0.77). The ReDO Death Score accounted for a significant interaction between age and Charlson score. It also accounted for lower predicted probability of death with lower baseline GFR. The ReDO Death Score had a median value (IQR) of 14 (10–17) and c-statistic of 0.65 (95% CI, 0.60 to 0.69). C-statistics for recovery and death in derivation cohort strata defined according to baseline eGFR are presented in Supplemental Table 5.

**Table 2:** ReDO Kidney Recovery Score

*Charlson*	**0**	**1**	**2**	**3**	**4**	**5**	**6**	**7**	**8**	**9**
	**−1**	**−2**	**−3**	**−4**	**−5**	**−6**	**−7**	**−8**	**−9**	**−10**
	**10**	**11**	**12**	**13**	**14**	**15**	**16**			
	−11	−12	−13	−14	−15	−16	−17			
*eGFR*	**0–2**	**3–4**	**5–7**	**8–9**	**10–12**	**13–14**	**15–17**	**18–19**	**20–22**	**23–24**
	4	21	29	34	38	42	44	47	48	50
	**25–27**	**28–29**	**30–32**	**33–34**	**35–37**	**38–39**	**40–42**	**43–44**	**45–47**	**48–49**
	52	53	55	56	57	58	59	60	61	62
	**50–52**	**53–54**	**55–57**	**58–59**	**60–62**	**63–64**	**65–67**	**68–69**	**70–72**	**73–74**
	62	63	64	65	65	66	67	67	68	68
	**75–77**	**78–79**	**80–82**	**83–84**	**85–87**	**88–89**	**90–92**	**93–94**	**95–97**	**98+**
	69	69	70	70	71	71	72	72	72	73
*Urine ACR*	**<3**	**3–30**	**>30**	**Not measured**						
	0	−2	−5	−5						
*Days from admission to 1st dialysis* *^a^*	**0–4**	**5–14**	**15–25**	**26–35**	**36+**					
	0	−1	−2	−3	−4					
*Dialysis**^a^* *first performed in ICU*	**Yes**									
	7									
*Days from first dialysis**^a^* *to discharge*	**0–2**	**3–8**	**9–15**	**16–22**	**23–28**	**29–35**	**36–42**	**43–48**	**49–55**	**56+**
	0	−1	−2	−3	−4	−5	−6	−7	−8	−9

The kidney recovery score is calculated by totaling the number of points for values of each covariate. The probability of recovery to dialysis independence was higher with higher scores. Italicized font indicates a model variable; boldface font indicates values for those variables; numbers below those values are the number of points attributed to that value. ReDO, recovery and death in outpatients; ACR, albumin-to-creatinine ratio; ICU, intensive care unit.

a Any form of KRT started during hospitalization.

**Table 3:** ReDO Death Score

***Cancer diagnosis (past 5 yr)***	**Lung**		**Breast**			**KUB**		**Myeloma**				**Other**	
	4		3			3		5				4	
***Discharge disposition***	**Indep**		**HC**		**NH**								
	0		2		3								
***eGFR***	**0–4**		**5–9**		**10–19**			**20–39**		**40+**			
	−14		−4		−2			−1		0			
***Days from first dialysis******^a^*** ***to discharge***	**≤3**		**4–9**		**10–39**			**40+**					
	0		1		2			3					
***Charlson***	**0**	**1**	**2**	**3**			**4**	**5**	**6**		**7**		**8**
* Age*													
18 to <30	0	2	4	5			7	9	11		12		14
30 to <40	1	3	4	6			7	9	10		12		14
40 to <50	3	4	6	7			8	10	11		12		13
50 to <60	5	6	7	8			10	11	12		13		14
60 to <70	8	9	10	10			11	12	13		14		15
70 to <80	11	12	12	13			14	14	15		15		16
80 to <90	15	15	16	16			16	17	17		18		18
90+	19	19	19	20			20	20	20		20		20
***Charlson***	**9**	**10**	**11**	**12**			**13**	**14**	**15**		**16**		
*Age*													
18 to <30	16	18	20	21			23	25	27		28		
30 to <40	15	17	18	20			21	23	24		26		
40 to <50	15	16	17	19			20	21	23		24		
50 to <60	15	16	17	18			19	20	21		22		
60 to <70	16	16	17	18			19	20	21		21		
70 to <80	17	17	18	18			19	20	20		21		
80 to <90	18	19	19	19			20	20	21		21		
90+	20	21	21	21			21	21	21		21		

The death score is calculated by totaling the number of points for values of each covariate. The probability of death was higher with higher scores. The Charlson Score is the sum of points for each baseline comorbidity present: one point: acute myocardial infarction, peripheral vascular disease, cerebrovascular disease, diabetes without complications, and hemiplegia/paraplegia; two points: congestive heart failure, chronic obstructive pulmonary disease, mild liver disease, diabetes with complications, and nonmetastatic cancer; three points: dementia and kidney disease; four points: nonmetastatic cancer, HIV disease; six points: metastatic cancer. Italicized font indicates a model variable; boldface font indicates values for those variables; numbers below those values are the number of points attributed to that value. ReDO, recovery and death in outpatients; KUB, kidney ureter or bladder; Indep, independence; HC, home care; NH, nursing home.

a Any form of KRT started during hospitalization.

**Figure 3:**
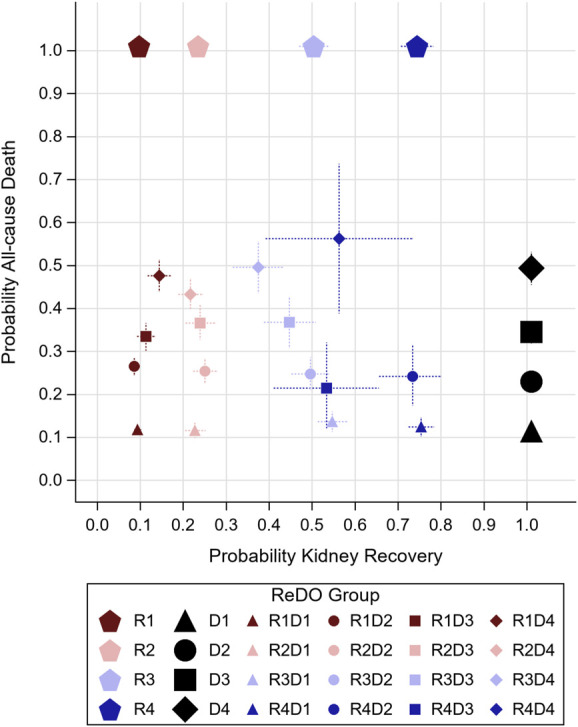
**Expected 1-year probability of kidney recovery to dialysis independence and death by the ReDO group.** Concurrent 1-year probabilities of death from any cause (vertical axis) and kidney recovery to dialysis independence (horizontal axis) are presented by the ReDO group. ReDO groups were created using the Recovery Score (Table 2) to determine the recovery quartile (R1: ≤32 points; R2: 33–47 points; R3: 48–59 points; R4: 60+ points) and the Death Score (Table 3) to determine the death quartile (D1: ≤14 points; D2: 15–17 points; D3: 18–19 points; D4: 20+ points). The 95% confidence intervals for both outcomes are presented around each group marker. Expected recovery risks for each recovery quartile are presented by different colored pentagons at the top of the figure. Expected death risks for each death quartile are presented by different black shapes at the right side of the figure. Data are also presented in Table 4.

When patients in the derivation cohort were allocated to one of 16 ReDO groups on the basis of their ReDO Recovery Score quartile and their ReDO Death Score quartile, ReDO groups demonstrated distinct 1-year probabilities of both outcomes (Figure 3). The probability of recovery to dialysis independence ranged from 10% (95% CI, 9% to 11%) in ReDO Group R1 to 73% (70% to 77%) in ReDO Group R4. The probability of death ranged from 12% (95% CI, 11% to 13%) in ReDO Group D1 to 46% (43% to 50%) in ReDO Group D4. When death and kidney recovery groups were considered concurrently, ReDO scores effectively segregated patients in the derivation cohort by event risks, with most groups having death and kidney recovery probabilities that were statistically distinct from other ReDO groups (Figure 3). With the exception of one group (R4D3), death risk and recovery probabilities were similar for ReDO categories within their corresponding death and recovery score quartiles, respectively.

Supplemental Figure 1 shows the observed versus expected probability of death or kidney recovery to dialysis independence for the derivation cohort.

### External Validation

In the validation cohort, the predicted probability of recovery to dialysis independence was significantly higher with successive ReDO Recovery Score quartiles (χ^2^ value 187.7, df=3, *P* < 0.0001), while the predicted probability of death was significantly higher with successive ReDO Death Score quartiles (χ^2^ value 92.3, df=3, *P* < 0.0001). The 1-year probabilities of recovery to dialysis independence and death by the ReDO group for both the derivation and validation cohorts are presented in Table 4.

**Figure 4:**
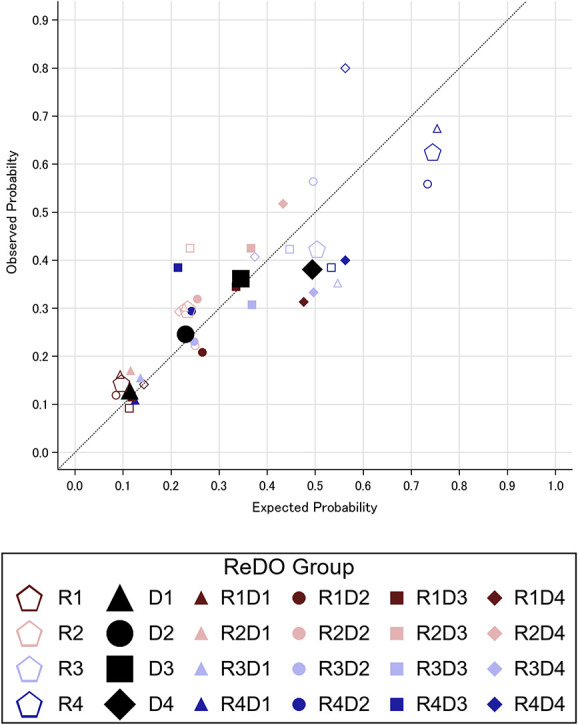
**Observed versus expected outcome probabilities in external validation.** This figure plots the observed outcome probability (vertical axis) against the expected outcome probability (horizontal axis). All patients in the external validation cohort were assigned to ReDO groups on the basis of their Recovery Score (Table 2) and their Death Score (Table 3). Expected probabilities for each group and outcome are from Figure 3, whose legend identifies each group above by marker shape and color. Recovery probabilities are presented with unfilled markers. Death probabilities are presented with filled markers. Markers for entire risk strata are larger than those for individual ReDO groups. Calibration slope and intercept for recovery were 0.76 and 0.07, respectively; for death, they were 0.76 and 0.04, respectively.

Figure 4 shows observed versus expected outcome probabilities for the external validation cohort. This is presented in more detail in Supplemental Table 6. The c-statistics (95% CI) for recovery and death quartiles were 0.70 (0.67 to 0.73) and 0.66 (0.62 to 0.69), respectively. ICI values (95% CI) for recovery and death quartiles were 7% (5% to 9%) and 4% (2% to 6%), respectively. Calibration slope and intercept for kidney recovery were 0.76 (95% CI, 0.54 to 0.98) and 0.07 (0.03 to 0.11), respectively; for death, they were 0.76 (95% CI, 0.58 to 0.93) and 0.04 (0 to 0.08), respectively.

## Discussion

We derived and externally validated risk indexes to predict the risks of kidney recovery to dialysis independence and death within 1 year for patients continuing outpatient dialysis after initiating dialysis in hospital. The models use data that are routinely available for hospitalized patients and apply to all those who start in-hospital dialysis (whether it be due to AKI, “AKI on CKD,” or progression of CKD to kidney failure) then continue dialysis as an outpatient. The probability-based risk groups can be used clinically to facilitate individualized prognostication and resource allocation for monitoring and follow-up care from the time of their first outpatient dialysis after hospitalization.

This work builds on prior knowledge related to the prediction of kidney function recovery after acute dialysis initiation. A study by Lee *et al.* used electronic medical records data of 2214 patients with AKI-D to derive a model for 90-day dialysis independence after starting dialysis in hospital (c-statistic 0.64).^23^ That study excluded patients with an expected in-patient risk of death >20%, did not account for the competing risk of death, and was not externally validated. Recovery to dialysis independence in that study was associated with higher baseline eGFR, less proteinuria, and less comorbidity at baseline, which is consistent with our findings.

A recent large retrospective study using United States Renal Data System data assessed kidney recovery to dialysis independence in children and adults starting dialysis for kidney failure as outpatients.^8^ This study, which did not include data on baseline kidney function, reported a 4% rate of recovery to dialysis independence within 1 year of initiation.^8^ All previous studies involving the same study population as ours (dialysis outpatients who started dialysis in hospital due to any cause) have been single-center studies,^5,7,24,25^ with the largest from Hickson *et al.*^5^ who reported on a retrospective cohort of 281 patients. Our finding that 19% of patients overall were dialysis-free at 1 year of discharge is nearly identical to the rate of recovery after a median of 8 months that was reported by that much smaller study*.*^5^

As confirmed by our kidney recovery model (Table 2, Supplemental Table 4), baseline eGFR is a strong predictor of recovery. Our results also provide confirmation of the importance of baseline proteinuria as a graded and independent predictor for kidney recovery, which has also been observed in previous studies of AKI^5,24,26^ and CKD progression.^27^ Notably, we found that patients were also less likely to be alive after 1 year when their baseline eGFR was higher. We surmise that this reflects the high mortality of acute illnesses causing severe AKI requiring dialysis in patients with previously preserved kidney function.

Our study has several strengths. First, we derived and externally validated our models specifically to assess outpatient kidney recovery. Second, the population-based data we used included important preadmission, intra-admission, and postadmission variables. Third, creating models for both death and recovery (and, crucially, accounting for the competing risk of death) increases the model's potential clinical utility because both likelihoods factor into patient decision making with respect to goals of care and planning longer-term treatment options for maintenance dialysis.

This study has several limitations. First, our definition of recovery to dialysis independence was not previously validated and may have included patients who stopped receiving dialysis as part of a strategy for conservative care in which the patient died >30 days after discontinuation of dialysis. This is an unlikely event given that, in Ontario, the median time to death after dialysis discontinuation is 4 (IQR, 2–6) days.^28^ Second, the lack of complete preadmission proteinuria (in particular, urine ACR) data is an important limitation, given the prognostic importance of proteinuria with respect to kidney recovery after AKI. The percentage of patients with available urine ACR data in the derivation cohort was also very high relative to the validation cohort and other studies.^26^ Third, we did not evaluate the models' performance using data beyond 2018, and coronavirus disease 2019–related changes in hospitalization patterns could affect their present day generalizability. Finally, and most importantly, while administrative data are useful for creating prediction models for relatively uncommon conditions, such as kidney failure necessitating initiation of dialysis in hospital, the lack of clinical data at the time of hospital admission and discharge is likely the most significant barrier to better model performance. The refinement of the ReDO model to incorporate primary data such as AKI cause and urine output^25^ before discharge might significantly improve the model's ability to predict recovery. Inclusion of primary data allowing for a determination of frailty might significantly improve the model's ability to predict death by more comprehensively capturing comorbidity severity after the acute illness that predicted hospitalization and the need to start dialysis.^29^

Our study is an important step toward better prognostication, with the ultimate aim of achieving better care for patients who require ongoing dialysis after hospitalization. We envision the ReDO models being used at the time of, or soon after, patients starting outpatient dialysis after hospitalization. This is a particularly relevant time to address care planning given that many outpatient dialysis facilities may not be well organized to otherwise identify and properly care for patients who are more likely to recover.^30^ Further refinement of the ReDO model to incorporate primary clinical data could allow for more precise identification of the patients most likely to benefit from interventions to optimize kidney recovery (*e.g.*, ReDO groups R3D1 and R4D1).^3,31,32^ Identification of these patients might also facilitate their inclusion into studies as the group most likely to benefit from interventions that promote kidney recovery. At the same time, patients determined to be likely to be alive but still requiring dialysis at 1 year (*e.g.*, ReDO groups R1D1, R2D1, or R1D2) could have their dialysis care optimized by expediting consideration of home therapies, arteriovenous access, and evaluation of transplantation. Another reason to revisit treatment decisions on transitioning to the outpatient setting is that patients starting dialysis in hospital are less likely to have had direct input into the decision to start dialysis in the context of AKI^33^ and/or other concurrent illness.^34^ At the time these patients transition to outpatient dialysis, they may be more likely to be able to provide direct input with respect to their wishes for ongoing treatment and with added understanding of what undergoing dialysis entails. Patients determined to be at high risk of death (*e.g.*, all D4—ReDO groups) could make better informed decisions regarding their overall goals of care with the additional understanding that recovery to dialysis independence is unlikely.

In conclusion, we derived and validated a risk index for predicting recovery of kidney function or death in the year after discharge from hospital for patients who continued to receive dialysis as an outpatient after initiating dialysis in hospital. An online tool on the basis of the ReDO models is available at https://qxmd.com/calculate/calculator_874.

**Table 4:** Observed 1-year probabilities of recovery to dialysis independence and death for recovery and death in outpatients groups in derivation and validation cohorts

ReDO Points		ReDO Groups	Derivation Cohort		Validation Cohort	
Recovery	Death		Recovery, % (95% CI)	Death, % (95% CI)	Recovery, % (95% CI)	Death, % (95% CI)
≤32	≤14	R1D1	9.4 (8.6 to 10.2)	11.8 (11.0 to 12.6)	16.2 (13.1 to 19.7)	11.5 (8.9 to 14.6)
	15–17	R1D2	7.5 (7.2 to 7.8)	26.6 (24.5 to 28.6)	11.9 (7.4 to 17.8)	20.8 (10.6 to 15.3)
	18–19	R1D3	11.0 (9.2 to 13.5)	33.4 (30.2 to 36.6)	9.2 (4.0 to 17.3)	34.5 (24.6 to 45.4)
	20+	R1D4	14.3 (11.9 to 17.1)	47.5 (43.8 to 51.3)	14.1 (8.0 to 22.6)	31.3 (22.4 to 41.4)
33–47	≤14	R2D1	22.8 (20.5 to 25.1)	11.6 (9.9 to 13.4)	30.2 (23.2 to 38.0)	17.0 (11.5 to 23.7)
	15–17	R2D2	25.1 (22.3 to 27.9)	25.5 (22.8 to 28.3)	22.2 (13.3 to 33.6)	31.9 (21.4 to 44.0)
	18–19	R2D3	24.0 (20.5 to 27.4)	36.7 (32.7 to 40.6)	42.5 (27.0 to 59.1)	42.5 (27.0 to 59.1)
	20+	R2D4	21.7 (18.8 to 24.5)	43.4 (39.8 to 46.8)	29.3 (18.1 to 42.7)	51.7 (38.2 to 65.1)
48–59	≤14	R3D1	54.8 (51.3 to 58.2)	13.5 (11.2 to 16.0)	35.2 (24.2 to 47.5)	15.5 (8.0 to 26.0)
	15–17	R3D2	49.6 (45.2 to 54.0)	24.8 (21.0 to 28.6)	56.4 (39.6 to 72.2)	23.1 (11.1 to 39.3)
	18–19	R3D3	44.8 (38.7 to 50.9)	36.8 (30.9 to 42.6)	42.3 (23.4 to 63.1)	30.1 (14.3 to 51.8)
	20+	R3D4	37.5 (31.6 to 43.2)	49.6 (43.6 to 55.8)	40.7 (22.4 to 61.2)	33.3 (16.5 to 54.0)
60+	≤14	R4D1	75.4 (72.4 to 78.2)	12.4 (10.0 to 14.8)	67.4 (56.8 to 76.8)	10.9 (5.3 to 19.1)
	15–17	R4D2	73.0 (65.7 to 80.0)	24.3 (17.4 to 31.4)	55.9 (37.9 to 72.8)	29.4 (15.1 to 47.5)
	18–19	R4D3	53.1 (40.6 to 65.5)	21.4 (11.7 to 32.2)	38.5 (13.9 to 68.4)	38.5 (13.9 to 68.4)
	20+	R4D4	56.2 (38.5 to 73.3)	56.2 (38.9 to 73.7)	80.0 (28.4 to 99.5)	40.0 (5.3 to 85.3)

For the derivation cohort, probabilities were generated using bootstrap sampling with confidence intervals using the percentile method. For the validation cohort, probabilities are those in each group with confidence intervals created using exact (*i.e.*, Clopper–Pearson) methods. ReDO, recovery and death in outpatients; CI, confidence interval.

## Data Availability

The analysis was conducted by members of the Institute of Clinical and Evaluative Sciences at the ICES uOttawa facility (Ottawa, Canada). The protocol can be obtained by emailing E.G. Clark at edclark@toh.ca.

## Supplemental Material

Supplemental Table 1. Datasets.

Supplemental Table 2. Diagnostic codes.

Supplemental Table 3. Outcomes according to subgroups for derivation and validation cohorts.

Supplemental Table 4. Kidney recovery and death models.

Supplemental Table 5. C-statistics for death and recovery in cohort strata defined according to baseline eGFR.

Supplemental Table 6. Observed versus expected risk of kidney recovery to dialysis independence or death in validation cohort.

Supplemental Figure 1. Calibration of ReDO Groups for 1-year death and kidney recovery to dialysis independence outcomes for derivation cohort.

Supplemental Material References.
